# Cardiac magnetic resonance imaging for post-ablation lesion assessment and guiding re-ablation strategies in patients with paroxysmal atrial fibrillation: Role of CMR in AF recurrence post-ablation

**DOI:** 10.1007/s10554-026-03655-3

**Published:** 2026-02-18

**Authors:** Sophie C. Rier, Tommaso Semino, Giulia De Zan, Marco Guglielmo, Hemanth Ramanna, Jeroen F. van der Heijden, Astrid A. Hendriks, Maarten J. Cramer, Pim van der Harst, Vincent J.H.M. van Driel, Ivo A.C. van der Bilt

**Affiliations:** 1 Department of Cardiology, Division of Heart and Lungs, Utrecht University, Utrecht University Medical Center, Utrecht, The Netherlands; 2 Department of Cardiology, Haga Teaching Hospital, Postbus 40551, Els Borst-Eilersplein 275, 2504 LN The Hague, The Netherlands; 3 Department of Medical Technology, The Hague University of Applied Sciences, Johanna Westerdijkplein 75, The Hague, 2521 EN The Netherlands

**Keywords:** Atrial fibrillation, Catheter ablation, Pulmonary vein isolation, Cardiac magnetic resonance, Atrial fibrosis, Arrhythmia recurrence

## Abstract

Atrial fibrillation (AF) recurrence after catheter ablation (CA), often due to incomplete lesions, remains a challenge. In this study, we evaluate the role of cardiac magnetic resonance (CMR) in characterizing lesion formation and guiding re-ablation strategies in patients with paroxysmal AF undergoing radiofrequency catheter ablation (RFCA). This prospective, single-center study enrolled patients undergoing first-time RFCA for paroxysmal AF. Pre-ablation CMR was performed for functional, anatomical, and fibrosis assessment. Repeat CMR 3 months post-ablation was performed to quantify fibrosis using Image Intensity Ratio (IIR) and detect lesion gaps, defined as ≥ 3 mm discontinuities in late gadolinium enhancement (LGE). AF recurrence was monitored for 18 months. 25 patients (20% female) were enrolled. 8/25 patients (32%) experienced AF recurrence. Post-ablation CMR showed significantly increased atrial fibrosis, though only in 2/25 patients (8%) complete circumferential pulmonary vein (PV) lesions were observed. Fibrosis burden did not differ significantly between patients with and without recurrence. Among 6 patients undergoing repeat ablation, 3 with LGE-defined gaps in circumferential lesions were successfully treated with a single re-isolation procedure. In contrast, 3 patients with extensive lesions and minimal or no gaps required multiple repeat procedures and additional ablation at extra-pulmonary vein sites. CMR can be a useful tool to assess post-ablation lesions and detect gaps after RFCA for paroxysmal AF. This may help distinguish recurrence due to insufficient initial ablation from other arrhythmogenic triggers, thereby guiding tailored and effective re-ablation strategies.

## Introduction

Atrial fibrillation (AF) represents the most common sustained cardiac arrhythmia globally, significantly impacting patient morbidity and mortality by increasing risks of stroke, heart failure, and reduced quality of life. Catheter ablation (CA), particularly pulmonary vein isolation (PVI), has emerged as a cornerstone in the management of symptomatic AF, serving as a first-line therapeutic option in selected patients [1–5]. PVI aims to create encircling lesions around the antra of the pulmonary veins to electrically isolate the left atrium from AF triggers originating in these veins. This procedure has demonstrated superior efficacy compared to antiarrhythmic drug therapy in maintaining sinus rhythm and alleviating symptoms [5–8]. 

Despite its established benefits, AF recurrence following CA remains a significant clinical challenge, with reported recurrence rates ranging broadly from 20% to 50% [9–13]. The primary mechanism underlying AF recurrence is frequently attributed to pulmonary vein (PV) reconnection [9]. This reconnection can be a consequence of acute, reversible myocardial cell injury and edema that initially renders lesions electrically effective, but then leads to reconnection once the cell injury and edema resolve [14]. While increased contact force during radiofrequency (RF) CA has been associated with increased controlled lesion formation [15], increased RF power and contact force are also associated with a higher rate of complications such as cardiac tamponade or esophageal damage [16]. Neither the reversibility of the lesion nor the development of complications can be monitored by fluoroscopy or electro-anatomical mapping, which are currently used to guide PVI [17]. 

Cardiac magnetic resonance (CMR) imaging offers superior soft-tissue contrast and high spatial resolution, making it an invaluable tool for detailed visualization of cardiac anatomy and tissue characterization. Its capabilities extend to quantifying pre-existing left atrial (LA) fibrosis, which has been associated with AF recurrence after the initial ablation procedure [18]. Furthermore, CMR can visualize early post-ablation wall thickening and edema, and crucially, identify gaps within ablation lesions that are implicated in PV reconnection and recurrence [19–22]. 

Despite these advantages, while CMR has demonstrated high sensitivity in detecting PV reconnection, its specificity in predicting actual PV reconnection or clinical AF recurrence is often noted as low [22]. Complete encirclement of the pulmonary veins by CMR-detected lesions is rare, with gaps found in up to 93% of patients. However, lesions that appear non-circumferential on CMR may still be electrically isolated, indicating that CMR may not fully capture the complete circumferential nature of the lesion [23, 24]. Actual recurrence of AF occurs much less frequently than the detection of gaps on CMR, indicating that a substantial number of patients maintain sinus rhythm after PVI despite incomplete encirclement of the pulmonary veins on CMR. Nevertheless, recurrence of AF is rare in those patients who do have complete encirclement of the pulmonary veins [20]. 

The primary aim of this study was to comprehensively assess PVI lesions using CMR imaging after RFCA in patients with new-onset paroxysmal AF. A secondary objective was to investigate the potential role of CMR in guiding management strategies for patients who experience recurrence of AF following their initial ablation procedure.

## Methods

### Study design and population

This was a prospective, single-center, observational study enrolling patients between June 2021 and November 2023 at Haga Teaching Hospital (The Hague, the Netherlands). Patients undergoing their first RFCA for new-onset paroxysmal AF were enrolled. Exclusion criteria included recent myocardial infarction, structural heart disease, prior cerebrovascular accident, severe renal impairment (eGFR ≤ 30 ml/min), and implantable devices. All enrolled patients provided written informed consent prior to participation, adhering to the local ethics committee guidelines.

Each patient underwent two CMR scans, the first pre-ablation and the second three months post-ablation. The timing of the second scan was chosen to minimize the confounding effects of edema and transient injury, which may lead to an overestimation of true fibrosis on acute post-procedural LGE CMR [25]. Clinical follow-up for AF recurrence was conducted over 18 months, with all adverse events documented throughout. Patients were evaluated at the outpatient clinic, where their history was taken regarding the return of symptoms, and an electrocardiogram registration was performed. Antiarrhythmic treatment was maintained until the patient’s first outpatient clinic visit, two weeks after the ablation. From that point, medication was tapered and discontinued based on the patient’s symptoms and the clinician’s judgment. Holter monitoring was performed at the clinician’s discretion. Patients who experienced recurrence of AF only during the blanking period (three months post-ablation) but not during the subsequent 15 months of follow-up were excluded from analysis in the recurrence group. As this was an observational study, CMR findings were not used to guide the management of AF recurrence, which was instead determined at the clinician’s discretion.

### Ablation procedure

We previously described the ablation procedure as performed in our centre [25]. In short, RFCA was performed under general anaesthesia using an electro-anatomical mapping system (Ensite NavX™, Abbot, St Paul, MN, USA). Circumferential point-by-point lesions were created with an irrigated tip catheter (Tacticath^®^, Abbott, St. Paul, MN, USA) around the pulmonary veins at the antral level. RF energy was applied with a maximum power of 30 W and a maximum electrode temperature of 43 °C. The Sensitherm Multi Esophageal Monitoring System (Abbott, St Paul, MN, USA) was used for esophageal temperature monitoring (ETM) to prevent esophageal injury during ablation. The cut-off temperature point was 39 °C. The procedural endpoint was confirmed as electrical isolation of all pulmonary veins, with conduction block rigorously verified through adenosine administration approximately 5 min after ablation of the right antrum and approximately 25 min after ablation of the left antrum.

### CMR imaging protocol

All CMR imaging was performed on a 1.5-T scanner (MAGNETOM Aera, Siemens Healthcare, Erlangen, Germany), with all scans conducted in sinus rhythm. During the scan before ablation detailed cardiac assessment was performed, including left ventricular and right ventricular volumes and function, LA volumes and function, baseline assessment of left atrial fibrosis, exclusion of left atrial appendage thrombus, and assessment of PV anatomy. The scan three months after ablation was used to evaluate chronic lesions, gaps in lesions, and post-ablation fibrosis. Localizer images and breath-hold steady state-free precession cine acquisitions were acquired. Cine acquisition sequence parameters were TR 3.54 ms, TE 1.16 ms, phases 25, flip-angle 80°, voxel 1,8 × 1,8 × 6.0 mm, slice thickness 6 mm. An ECG-gated free-breathing three-dimensional (3D) contrast-enhanced angiography of the left atrium and pulmonary veins was performed. Sequence parameters were TR 3.97 ms, TE 1.54 ms, bandwidth 496 Hz/pixel, echo spacing 3.5 ms, voxel 0.6 × 0.6 × 1.3 mm, slice thickness 1.25 mm, TI 150 ms, flip angle 20°. Left atrial late gadolinium enhancement (LGE) images were acquired using ECG-triggered, free-breathing respiration-navigator-gated three-dimensional (3D) whole heart imaging 20 min after injection of 0.1 mmol/kg gadolinium contrast agent (Dotarem 0.5 mmol/ml) using inversion-recovery prepared protocols. 3D-LGE sequence parameters were: field of view 360 × 360 × 110 mm, echo spacing 4.90, echo time 2.09 ms, bandwidth 271 Hz/pixel, voxel 0.6 × 0.6 × 2.5 mm, flip angle 19°. Inversion time was selected using a dedicated TI scout sequence to null healthy myocardium and was between 250 and 380 ms.

### CMR imaging post-processing

LA fibrosis was assessed using the 3D LGE sequence. Post-processing was performed using ADAS 3D software (Adas3D Medical SL, Barcelona, Spain) as previously described [22]. After manual tracing of the left atrial wall in multiple axial plane slices, ADAS 3D was used to automatically create a 3D model of the left atrium, with manual adjustment when necessary. LGE fibrosis was quantitatively assessed using the image intensity ratio (IIR) method as previously described [26]. In short, the signal intensity of the left atrial wall is divided by the mean signal intensity of the entire left atrial blood pool, with an IIR > 1.20 defining atrial interstitial fibrosis and an IIR > 1.32 defining dense scar [27, 28]. Image intensity ratios were color-coded and projected into the 3D model. Discontinuity of LGE ≥ mm 3 was considered a gap as previously described [22, 29]. The pulmonary veins and mitral valve were excluded from analysis.

### Statistics

All statistical analyses were performed using Jamovi software, with significance set at *P* < 0.05. Continuous variables are expressed as mean and standard deviation (SD) when normal or as median and interquartile range (IQR) when not normal. Normality was assessed through the Shapiro-Wilk test. Continuous quantitative data were compared through a t-test, or Mann-Whitney U if a non-parametric test was required. Categorical data are expressed as percentages and were compared through Chi-squared tests.

## Results

### Baseline characteristics, left atrium, and left ventricle parameters

25 patients (20% female) were enrolled. There were no statistically significant differences in baseline characteristics (Table 1) or left atrium and left ventricle parameters (Table 2) between patients who had recurrence of AF during the follow-up period and patients who did not. All patients had little to no left atrial fibrosis at baseline, and would be classified as stage 1 atrial fibrosis [18]. 

**Table 1:** Baseline characteristics

Patients characteristics	Total (*N* = 25, 100%)	No recurrence (*N* = 17, 68%)	Recurrence (*N* = 8, 32%)	*P*-value*
Age	62.1 ± 6.3	61 ± 6.2	64.5 ± 5.6	0.37
Female sex	5 (20%)	3 (18%)	2 (25%)	0.67
Body mass index (kg/m2)	26.9 ± 3.7	26.8 ± 3.4	27.17 ± 3.8	0.73
Hypertension	9 (36%)	6 (35%)	3 (38%)	0.92
Diabetes mellitus	1 (4%)	1 (6%)	0	0.48
Dyslipidemia	3 (12%)	3 (18%)	0	0.21
CHADS2VA	0.9 ± 0.9	1.1 ± 0.9	0.9 ± 0.9	0.67
Medical therapy				
Beta-blockers other than Sotalol Digoxin Calcium blockers	5 (20%) 1 (4%) 3 (12%)	4 (24%) 0 1 (6%)	1 (13%) 1 (13%) 2 (25%)	0.52 0.14 0.17
Flecainide	3 (12%)	2 (12%)	1 (13%)	0.96
Sotalol	12 (48%)	8 (47%)	4 (50%)	0.89
Amiodarone	2 (8%)	1 (6%)	1 (13%)	0.57

* P-value assessing the difference between patients with recurrence and those without recurrence

**Table 2:** Left atrium and left ventricle parameters

	Total (N=25, 100%)	No recurrence (N=17, 68%)	Recurrence (N=8, 32%)	P-value*
LAV (ml)	87.3 ± 26.0	83.1 ± 23.7	96.2 ± 28.5	0.26
LAVI (ml/m2)	41.1 ± 11.3	39.6 ± 10.6	44.4 ± 12.0	0.41
LV EDV	167.3 ± 33.1	167.6 ± 32.3	166.6 ± 34.8	1.00
LV ESV	67.4 ± 17.3	68.6 ± 16.7	65.0 ± 18.09	0.67
LV EF	59.8 ± 5.9	59.1 ± 5.6	61.2 ± 6.5	0.51

* P-value assessing the difference between patients with recurrence and those without recurrence LAV: left atrial volume; LAVI: left atrial volume index; LV EDV: left ventricular end-diastolic volume; LV ESV: left ventricular end-systolic volume; LV EF: left ventricular ejection fraction

### Clinical follow-up

During the 18-month follow-up period, 8 out of the 25 enrolled patients (32%) experienced a recurrence of AF after the blanking period of 3 months. Of these 8 patients, 2 had a single, short episode of AF recurrence that spontaneously terminated without the need for medication or electrocardioversion. The remaining 6 patients (24%), who experienced sustained or symptomatic recurrences, subsequently underwent a re-ablation procedure. No embolic events suggestive of undetected atrial fibrillation recurrence occurred.

### Assessment and quantification of LGE

**Fig. 1:**
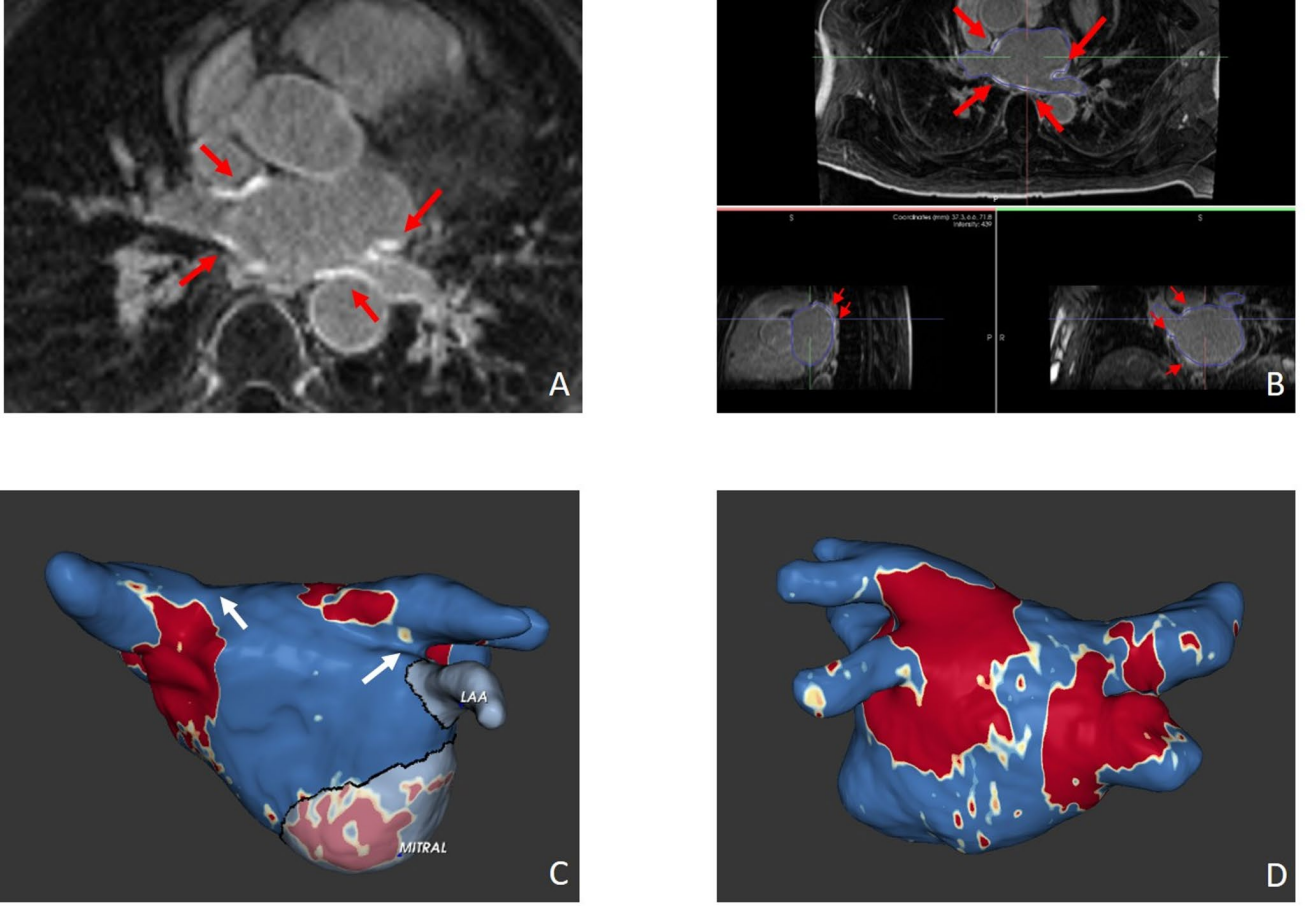
Representative case of atrial fibrosis assessment

Only in 2 out of 25 patients (8%) completely circumferential lesions were observed around the PVs without gaps at the antral level after ablation. Figure 1 shows a representative example of atrial fibrosis assessment. Table 3 shows the percentage of LA fibrosis before and after ablation. There was no significant difference in the amount of fibrosis before or after ablation between patients who had recurrence of AF and patients who did not.

(A) Screenshot taken in Circle CVI^42^. Axial view of 3D LGE image with enhancement in the ablation areas indicated by red arrows. B-D Screenshots taken in ADAS 3D. (B) Segmentation of the left atrial wall with enhancement in ablation areas in different views indicated by red arrows. (C) Anterior view of 3D model with color-coding of image intensity ratio, with blue areas representing healthy myocardium and red areas representing fibrosis. In this patient, gaps can be seen anterior to the left pulmonary veins and in the roof above the right superior pulmonary vein, indicated by white arrows. (D) Posterior view of the 3D model with more extensive lesions compared to the anterior view.

**Table 3:** Percentage of fibrosis of the left atrial wall

Percentage LGE before ablation	Total (*N* = 25)	No recurrence (*N* = 17, 68%)	Recurrence (*N* = 8, 32%)	*P*-value*
	4.1% ± 3.9%	3.9% ± 3.4%	4.4% ± 4.7%	0.71
Percentage LGE after ablation	16.3% ± 7.6%	16.6% ± 6.0%	15.6% ± 10.1%	0.59

* P-value assessing the difference between patients with recurrence and those without recurrence

### Correlation between gaps in LGE and the success of re-ablation

**Fig. 2:**
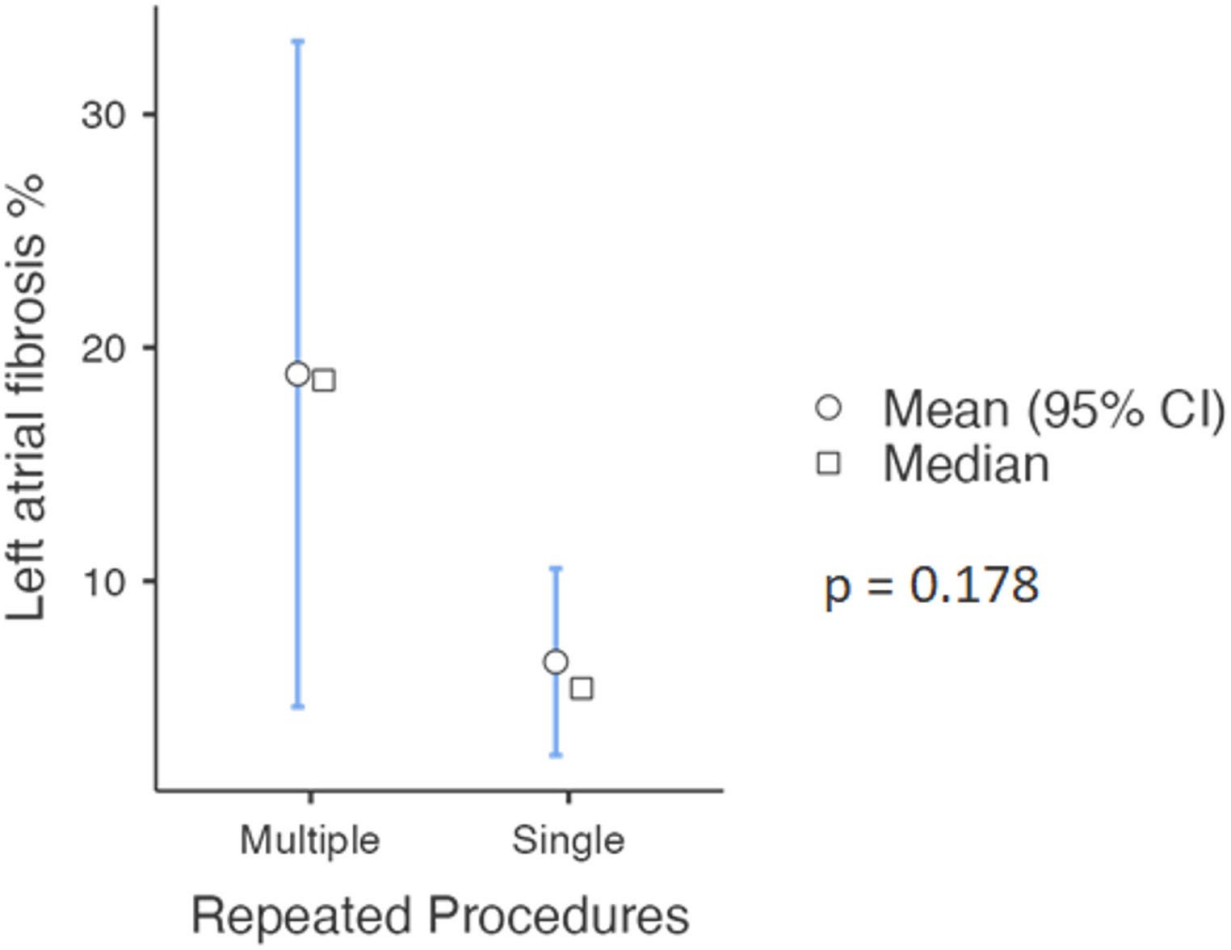
Percentage of atrial fibrosis in patients with recurrence of AF requiring multiple or only a single repeat ablation

Of the 6 patients who experienced AF recurrence and subsequently underwent re-ablation, CMR provided distinct findings. Three of these patients presented with clear, identifiable gaps in the continuous circular lesions around the PVs. The other 3 patients, in contrast, showed extensive lesions around the PVs with minimal and in one case no gaps. Although this intriguing difference was reflected in the percentage of atrial scar (Table 4; Fig. 2), the statistical power was insufficient, preventing it from reaching statistical significance. The 3 patients with clear gaps in LGE lesions were all treated by re-isolating the pulmonary vein antra, and no additional ablation, such as ablation of the posterior wall, was performed. All three remained free of AF after this single re-do procedure for the remainder of the follow-up period. Conversely, the 3 patients who had little or no gaps were not free of AF after one re-do procedure. All of these patients required at least two repeat procedures during the remainder of the follow-up period and still experienced recurrence afterwards. In one of these 3 patients, both antra were completely encircled on CMR imaging, and the pulmonary veins were still isolated upon re-procedure, and voltage mapping showed no low voltage areas, leading to ablation of the posterior wall being performed. The second patient was in AF during the re-procedure and had two small reconnections in the roof; AF persisted after an attempt at electrocardioversion, and even after re-isolating the pulmonary veins, with termination only achieved after performing ablation of the posterior wall. Adequate voltage mapping was not possible due to the atrial fibrillation. In the third patient, the left antrum was isolated upon re-procedure, as was the right superior pulmonary vein, with only a small leak at the right inferior pulmonary vein. Besides re-isolating the pulmonary veins, debulking of fractionated areas septal near the right superior pulmonary vein and near the left inferior pulmonary vein was performed.

**Table 4:** Percentage fibrosis of the left atrial wall and treatment during re-procedure in patients with recurrence

Percentage LGE after ablation	Recurrence successfully treated with one re-procedure (*N* = 3)	Recurrence requiring more than one re-procedure (*N* = 3)
	6.6% ± 3.5%*	18.9% ± 12.6%*
Treatment during re-procedure	All patients: treatment of several reconnections in the pulmonary vein antra, no additional ablation	Patient 1: pulmonary veins already isolated during re-procedure, posterior wall ablation performed Patient 2: two small reconnections around pulmonary veins treated, but termination of AF only after posterior wall ablation Patient 3: small leak at RIPV treated, debulking of fractionated areas

* P-value assessing the difference between patients with recurrence and those without recurrence = 0.18

## Discussion

The present study demonstrates the utility of CMR imaging in assessing post-ablation lesions and a potential role in guiding re-ablation strategies in patients experiencing AF recurrence after an initial RFCA.

Patients who are free of AF recurrence after PVI may still have gaps in the continuous circular lesions. The question remains whether the incomplete lesion formation in the antra is based on actual gaps in ablation lines or whether it is actually related to the low specificity of CMR in detecting PV reconnection. The observation that only 8% (2 out of 25) of patients achieved completely encircled PV lesions after ablation is consistent with previous research, which reported similar rates of 7–11% [22–24]. Despite this low rate of complete encirclement, a substantial number of patients with CMR-detected gaps did not experience AF recurrence, and no significant difference in the amount of LGE was found between patients with and without AF recurrence. This finding supports the notion that not all gaps visualized by CMR are electrophysiologically or clinically significant, which aligns with the reported low specificity of CMR in predicting PV reconnection [22]. 

Failure of catheter ablation for AF is generally caused by incomplete treatment of the intended target(s), which may be seen as a technical failure, or by incomplete selection of ablation target(s), which may be seen as a pathophysiological failure. A key finding in this study is the ability of CMR to identify patients who experienced recurrence of AF despite having largely intact continuous circular lesions on CMR and who had limited benefit from a repeat procedure. These are probably recurrences due to other pathophysiological mechanisms such as extra-pulmonary triggers and substrate. Although this information can be obtained through electroanatomic mapping, CMR offers a non-invasive alternative.

Conversely, CMR also identified patients who recurred with clear gaps in their PV lesions, who subsequently benefited from targeted re-isolation of the PVs during a single repeat procedure. These recurrences seem to be clearly related to insufficient lesion formation in the continuous circular lesions around the PVs from the index ablation procedure, requiring re-ablation to repair and perfect the previous intended work.

**Fig. 3:**
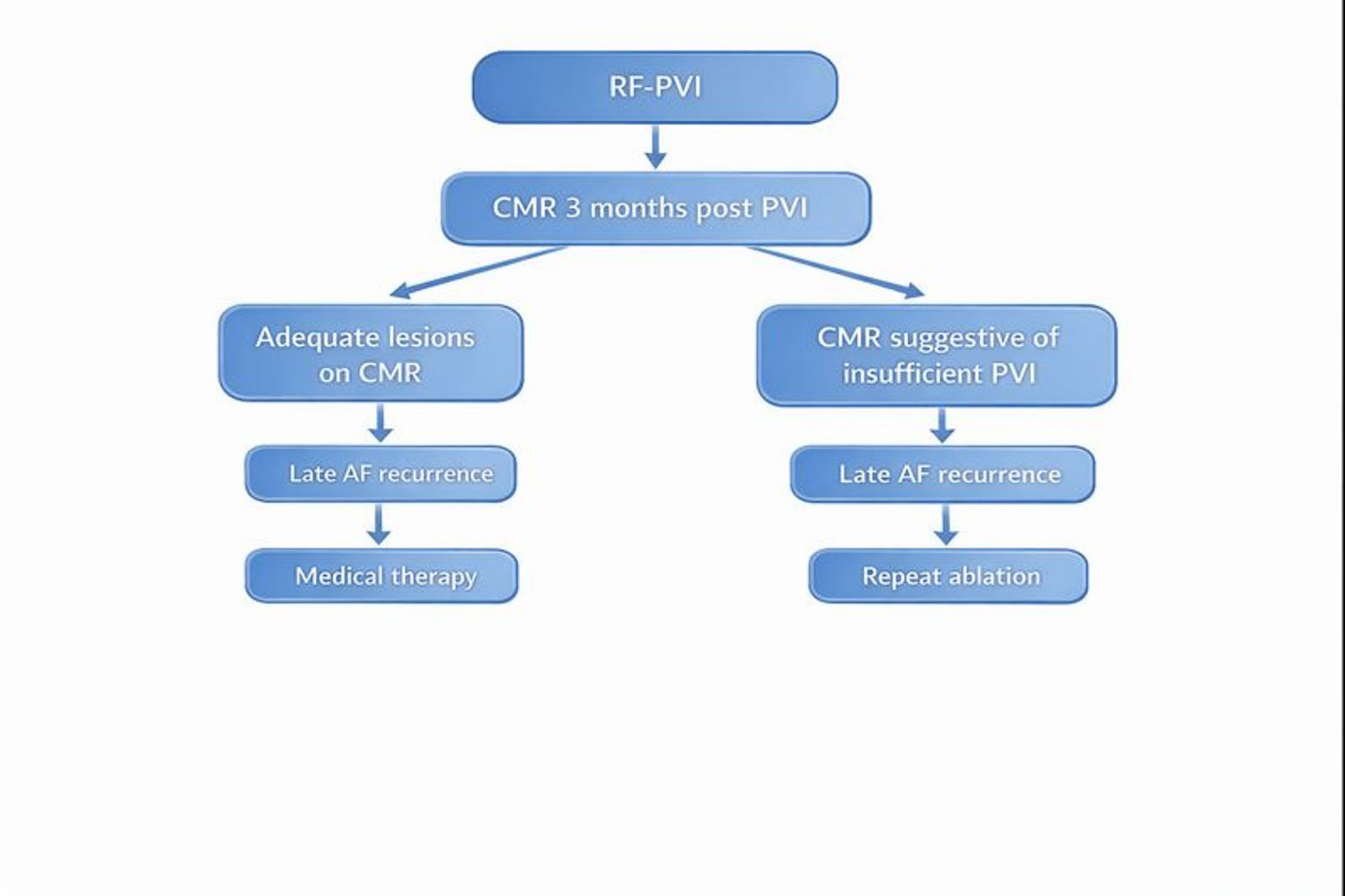
Proposed study design for a prospective trial to evaluate the efficacy of CMR-guided re-ablation in managing atrial fibrillation recurrence following radiofrequency catheter ablation

These findings suggest that CMR could be a valuable tool in distinguishing between recurrences caused by insufficient initial ablation and those originating from other arrhythmogenic triggers and substrate outside the pulmonary veins, thereby enabling a more targeted re-ablation strategy. However, as this was an observational study in which CMR was not used to guide clinical management, these findings are explorative rather than definitive. A prospective experimental study in which re-ablation is guided by CMR could further validate its potential in shaping re-ablation strategies (Fig. 3).

Several limitations have to be considered regarding these findings. First, the study was conducted at a single center and involved a relatively small cohort of 25 patients, which limits the statistical power and generalizability of the results. The study’s focus on new-onset paroxysmal AF patients undergoing their first RFCA, while enhancing internal validity by minimizing confounding factors related to advanced atrial disease or prior interventions, also restricts the direct applicability of these findings to more complex AF phenotypes or patient populations.

Second, Holter monitoring was performed based on the clinician’s discretion, rather than as a routine procedure. This could have resulted in an underdetection of asymptomatic AF episodes, potentially leading to a reported recurrence rate lower than the true recurrence rate.

Third, while post-processing of LGE CMR of the left atrium using the IIR method with ADAS 3D software has demonstrated reproducibility [27, 28], the acquisition of 3D LGE sequences can vary among centers. Factors such as the timing of scanning after contrast administration, the contrast dose, and patient-specific factors (e.g., body mass index, renal function, hematocrit) can influence the identified percentage of post-ablation atrial scar and the signal-to-noise ratio [30]. The timing of CMR post-ablation is particularly relevant; as demonstrated by Althoff et al., LGE CMR most accurately detects definite lesion formation at 3 months post-ablation, with its ability to detect fibrosis decreasing significantly after 12 months [29]. Implementing this approach would require routine CMR imaging three months after ablation, ensuring that the most accurately detected lesions are available in the event of a recurrence. While this could increase costs and pose practical challenges, it may ultimately reduce the need for unnecessary repeat procedures, offering potential cost savings in the long term.

Fourth, the study exclusively utilized radiofrequency ablation. Regany-Closa et al. [31] recently described how different ablation techniques, such as cryoballoon ablation, pulsed-field ablation, or high-power short-duration RF ablation, produce distinct lesion characteristics and durability profiles. Therefore, the relationship between CMR-detected lesions/gaps and clinical outcomes established in this RFCA-specific study may not be directly applicable to patients treated with other modalities.

Finally, LA strain measurements were not included in this analysis. This should be considered in future studies because LA reservoir strain, especially, may be useful in detecting subtle LA dysfunction that might influence procedural success rates [32]. 

Despite these limitations, the study’s findings provide valuable information regarding the clinical utility of CMR in guiding repeat ablation procedures. The ability to differentiate between recurrences due to anatomical gaps (suggesting insufficient initial ablation) and those due to other triggers (requiring broader ablation strategies) has significant implications for personalized patient management. CMR performed three months after ablation can help clarify this distinction and guide decisions on whether a patient with AF recurrence would likely benefit from targeted re-treatment of the pulmonary veins, or if other underlying triggers are more probable, in which case a repeat ablation may be less beneficial. A prospective trial evaluating this workflow would be a valuable next step in validating the clinical utility of CMR for guiding repeat ablation procedures.

## Conclusion

Following RFCA for paroxysmal AF, CMR imaging can play a useful role in assessing post-ablation lesions. This capability is significant as it can help guide the selection of patients with AF recurrence who are most likely to benefit from a repeat procedure. Specifically, in patients with recurrence but no significant gaps on CMR, the presence of other arrhythmogenic triggers is suggested, necessitating more extensive ablation approaches and potentially multiple repeat procedures. This finding facilitates a more tailored and efficient approach to managing AF recurrence, which potentially optimizes procedural outcomes and anticipates on reducing the burden of repeat interventions. A prospective, experimental study with a larger sample size, where re-ablation is guided by CMR, could further validate this potential.

## Data Availability

The data that support the findings of this study are not openly available due to reasons of sensitivity and are available from the corresponding author upon reasonable request. Data are located in controlled access data storage at Haga Teaching Hospital.

## Abbreviations

AF: Atrial fibrillation
CA: Catheter ablation
CMR: Cardiac magnetic resonance
IIR: Image intensity ratio
LA: Left atrium
LGE: Late gadolinium enhancement
PV: Pulmonary vein
PVI: Pulmonary vein isolation
RF: Radiofrequency
RFCA: Radiofrequency catheter ablation
